# De‐diagnosing chronic lymphocytic leukaemia: An ethical and scientific case for changing diagnostic criteria

**DOI:** 10.1002/hem3.70252

**Published:** 2025-11-19

**Authors:** Peter Johnstone, Peter Allen, Pinky Jimenez‐Agrawal, Samir Agrawal, Stephen P. Hibbs

**Affiliations:** ^1^ Barts Cancer Institute Queen Mary University of London London United Kingdom; ^2^ CLL Support Association Chippenham United Kingdom; ^3^ Ichor Health Kent United Kingdom; ^4^ Centre for Immunobiology and Infection, Blizard Institute Queen Mary University of London London United Kingdom; ^5^ Department of Haemato‐Oncology Barts Cancer Centre, St Bartholomew's Hospital London United Kingdom; ^6^ Wolfson Institute of Population Health Queen Mary University of London London United Kingdom

Overdiagnosis describes situations where ‘people are labelled with or treated for a disease that would never cause them harm’.^1^ We believe the current diagnostic paradigm for chronic lymphocytic leukaemia (CLL) constitutes overdiagnosis, imposing a diagnosis which can cause significant distress to patients without benefit. In this article, we begin with the personal experience of one of the authors (Peter Allen), which powerfully depicts the harms of a CLL label. We then consider the historical context of current CLL diagnostic criteria and question their suitability. Finally, we consider other possible approaches to CLL diagnosis, including intentional non‐investigation and de‐diagnosis.

## This is what ‘watch and wait’ does (Peter Allen)

In 2001, I was 30 years old, fit and healthy, with no reason to think anything was wrong. A routine workplace health check changed everything. My blood results showed a lymphocyte count of 8.0 × 10^9^/L. More tests followed, then a bone marrow biopsy. The doctor told me I had CLL. I was placed on ‘Watch and Wait’ and for the last 24 years I've not had any symptoms. I had to go home and carry on life, knowing I had leukaemia but no treatment.

In those early months, I looked for ways to help myself. Diet, exercise, anything. My consultant told me none of it would make a difference to the leukaemia. I felt helpless. My motivation disappeared, and I put on a lot of weight. My future became a fog.

The uncertainty was crushing. My sports and hobbies were suddenly ‘too risky’. I was even told to avoid time in the sun, for risk of second malignancies. Yet in the same breath, my doctor told me to ‘live normally’.

The projections doctors gave me were based on much older patients. I was told that I might need a stem‐cell transplant, or I might die. That prediction hung over me like a clock ticking down. In 2003, a ZAP‐70 test brought good news: my CLL was likely to progress slowly. As a result, my partner and I decided to have another child.

Work was another battle. At first, my employer was supportive. Then things changed. I could feel colleagues treating me differently, assuming I wouldn't aim for promotions. I stopped telling future employers about my diagnosis. Even insurance became challenging: travel and life cover were almost impossible to get, limiting what I could do. By 2006, I suffered from anxiety and depression. I tried cognitive behavioural therapy, but it didn't help. Years later, I found some peace through yoga and mindfulness.

Looking back, the label ‘CLL’ carried more weight than my actual condition. It didn't reflect the complexity or uncertainty of what I had. Outside the hospital, the word ‘cancer’ conveyed fear, assumptions and misconceptions. Over time, I learned to turn that fear into action. I got involved in charity work and research, and I'm now Vice‐Chair of the UK CLL Support Association.

My CLL has neither disappeared nor required treatment. After 25 years, I've been discharged from regular testing. I'm grateful for my outcome, but I'll always wonder if I could have been spared the diagnosis and psychological and practical burdens that the label has brought.

## Questioning current diagnostic criteria

How do we make sense of Peter's diagnosis and experience? CLL is typically a slowly progressive condition with a median survival time of approximately 11 years, though this can be significantly longer for those with low‐risk disease and minimal comorbidities.^2^, ^3^ The current diagnostic threshold is a clonal B cell count of >5 × 10^9^/L,^4^ but patients will typically reach much higher counts before they become symptomatic. Historically, CLL was a disease of the elderly. However, with widespread blood tests and increased access to diagnostic flow cytometry, both the median age at diagnosis and the median lymphocyte count at diagnosis are gradually decreasing.^5^

As a result, approximately 80% of patients are now low‐risk Binet Stage A at diagnosis^3^ and are managed with a surveillance or ‘watch and wait’ strategy. Many of these patients remain under surveillance for years—75%–90% of patients with a low‐risk CLL‐IPI score will be treatment free at 5 years, whilst their risk of death attributable to CLL is the same as their risk of death from other causes.^6^, ^7^, ^8^

It is this group of patients, with asymptomatic early‐stage CLL, who we believe are being poorly served by the current approach. As Peter's account shows, the cognitive dissonance is extreme: individuals are given a label of an ‘incurable leukaemia’ but informed no cancer‐directed treatment will be offered until progression.^9^ The deleterious effects of a cancer diagnosis on quality of life are well documented.^10^, ^11^ CLL patients have been shown to have high levels of anxiety and emotional distress. Importantly, this reduced quality of life does not improve over time for CLL patients under active surveillance, suggesting psychological impacts are not relieved by reassurance from a clinician.^12^, ^13^

The implicit assumption of current diagnostic criteria is that the benefits of diagnosis outweigh these negative impacts. The benefits of any diagnosis can be divided in three broad categories: providing a patient with an explanation for their symptoms; accessing healthcare services for management of these symptoms and any underlying disease; and/or identifying an abnormality that can be treated prior to symptomatic disease. We argue that few if any of these benefits are present in the diagnosis of early‐stage asymptomatic CLL.

By definition, in asymptomatic disease, there are no symptoms to either explain or treat. In other comparable diseases such as high‐risk MGUS, there may be a benefit to diagnosis to enable early treatment before symptoms^14^; however, there is no evidence of benefit in treating early‐stage asymptomatic CLL.^15^, ^16^

Does regular surveillance of CLL provide benefit? Guidelines suggest regular 6–12 monthly specialist review,^17^ to look for symptoms or a change in the lymphocyte count, which could suggest progression. However, most low‐risk patients will still be treatment free at 5 years. Appointments are a significant burden on the resources of both the individual patients and the healthcare system. For many patients having repeated healthcare appointments where no intervention is carried out only increases their anxiety.

A recent study demonstrated that patients scoring as low risk on the ‘CLL‐WONT’ score and low risk on the CLL‐IPI score could safely have their follow‐up moved to non‐specialist primary care providers with no impact on outcomes.^18^ Whilst this strategy reduces unnecessary specialist follow‐up, it also creates the paradoxical situation of diagnosing a patient with ‘leukaemia’ but advising management with their primary care doctor—a further example of incongruence between the diagnostic label and the context of disease management.

Not only does the current diagnostic approach to CLL gloss over the damage of the label, it also has limited contemporary clinical relevance. Historically, diagnostic criteria were based on lymphocyte counts as the most easily tested factor, but this approach began when most patients presented symptomatically with lymphocyte counts significantly above the diagnostic threshold and hence the threshold itself mattered less. Nowadays, far more patients are diagnosed incidentally with relatively low lymphocyte counts, but clinicians continue to use an arbitrary cut‐off of a B cell count of >5 × 10^9^/L. This diagnostic lymphocyte threshold persists despite poorly predicting progression or survival. Interestingly, when the CLL‐IPI prognostic score was used to look at both patients with MBL and low‐count CLL, patients who were labelled as MBL but high risk on the CLL‐IPI score had a shorter time to first treatment and overall survival than patients diagnosed as CLL but low‐risk on the CLL‐IPI score.^8^ This contradiction further underlines the fact that the current arbitrary threshold between the two entities is not fit for purpose.

## What other diagnostic approaches are possible?

How, then, should diagnostic practice change? Clearly, in patients with symptoms or cytopenias that would trigger a decision for CLL‐directed treatment, diagnosis serves a clear purpose. The most radical option would be to reserve the diagnostic label of CLL for only those needing treatment. However, for individuals who are very likely to need treatment, the label will also serve some utility in helping someone to make plans and prepare themselves.

Instead, we would advocate changing the CLL diagnostic threshold to a clonal B cell count of >10 × 10^9^/L—this is a better predictor of overall survival and time to first treatment.^19^ This change would recategorise many newly diagnosed patients as MBL, reducing some of the impact of a ‘leukaemia’ diagnostic label. Furthermore, in many cases, this would allow clinicians to ‘de‐diagnose’ some individual patients, removing the CLL label. De‐diagnosis is the process of ‘removing diagnoses that do not contribute to the reduction of persons' suffering’.^20^ However, a nomenclature switch from CLL to MBL only partially removes the damage from unnecessary overmedicalisation: the otherwise healthy individual remains a patient, albeit with a less harmful label.^21^

Therefore, increasing the diagnostic threshold should be part of a broader effort to approaching the diagnosis of asymptomatic lymphocytosis. This should address the central question: ‘how can the diagnostic and conceptual approach to asymptomatic clonal lymphocytosis be ethically reimagined based on the needs of those most affected?’ and should involve those diagnosed with asymptomatic CLL and MBL, members of the wider public, ethicists, haematologists and primary care doctors. The thought of *not investigating* clonal lymphocytosis sits uncomfortably with many clinicians, but is common in other areas of medicine, such as many incidental radiological findings (e.g., small thyroid nodules) or withholding hereditary thrombophilia investigations in most individuals with venous thrombosis. As the ability to detect deviations from the norm grows ever greater, we must remember that labelling and monitoring such deviations is only sometimes helpful.

We envisage that this process of reimagining could include work to better identify the lowest risk prognostic groups who could be safely discharged without a diagnostic label; work with patient groups to identify those who would benefit from ‘de‐diagnosis’ and consider how this process might look from a patient perspective; and work with haematologists and primary care providers to establish a workable and ethical framework of *intentional non‐investigation* for some individuals with asymptomatic lymphocytosis.^22^ The current approach to CLL diagnosis was pragmatically *constructed* by clinicians rather than reflecting absolute scientific truths. Now, clinicians must collectively take the brave step of changing diagnostic criteria to fix the harms caused by CLL overdiagnosis.

## AUTHOR CONTRIBUTIONS

**Peter Johnstone**: Conceptualisation; writing—original draft; writing—review and editing; project administration. **Peter Allen**: Writing—review and editing; writing—original draft. **Pinky Jimenez‐Agrawal**: Writing—review and editing. **Samir Agrawal**: Writing—review and editing. **Stephen P. Hibbs**: Conceptualization; writing—original draft; project administration; writing—review and editing.

## CONFLICT OF INTEREST STATEMENT

Pinky Jimenez‐Agrawal reports speaker engagements with AstraZeneca, BeOne (formerly BeiGene) and Eli Lilly. Samir Agrawal reports speaker engagements with AbbVie, BeOne (formerly BeiGene) and AstraZeneca.

The remaining authors (P.J., P.A. and S.P.H.) have no relevant conflicts of interest to declare.

## FUNDING

S.P.H. is supported by a HARP doctoral research fellowship, funded by the Wellcome Trust (Grant number 223500/Z/21/Z).

## Data Availability

Data sharing is not applicable to this article as no datasets were generated or analysed during the current study.

## References

[1] Pathirana T , Clark J , Moynihan R . Mapping the drivers of overdiagnosis to potential solutions. BMJ. 2017;358:j3879. 28814436 10.1136/bmj.j3879

[2] Schlosser P , Schiwitza A , Klaus J , et al. Conditional survival to assess prognosis in patients with chronic lymphocytic leukemia. Ann Hematol. 2024;103:1613‐1622. 38308707 10.1007/s00277-024-05627-w PMC11009732

[3] Weide R , Feiten S , Chakupurakal G , et al. Survival improvement of patients with chronic lymphocytic leukemia (CLL) in routine care 1995–2017. Leuk Lymphoma. 2020;61:557‐566. 31682164 10.1080/10428194.2019.1680840

[4] Hallek M , Cheson BD , Catovsky D , et al. iwCLL guidelines for diagnosis, indications for treatment, response assessment, and supportive management of CLL. Blood. 2018;131:2745‐2760. 29540348 10.1182/blood-2017-09-806398

[5] Abrisqueta P , Pereira A , Rozman C , et al. Improving survival in patients with chronic lymphocytic leukemia (1980–2008): the Hospital Clínic of Barcelona experience. Blood. 2009;114:2044‐2050. 19553638 10.1182/blood-2009-04-214346

[6] Molica S , Shanafelt TD , Giannarelli D , et al. The chronic lymphocytic leukemia international prognostic index predicts time to first treatment in early CLL: independent validation in a prospective cohort of early stage patients. Am J Hematol. 2016;91:1090‐1095. 27465919 10.1002/ajh.24493 PMC5072993

[7] An international prognostic index for patients with chronic lymphocytic leukaemia (CLL‐IPI): a meta‐analysis of individual patient data. *Lancet Oncol*. 2016;17:779‐790. 10.1016/S1470-2045(16)30029-8 27185642

[8] Parikh SA , Rabe KG , Kay NE , et al. The CLL International Prognostic Index predicts outcomes in monoclonal B‐cell lymphocytosis and Rai 0 CLL. Blood. 2021;138:149‐159. 33876228 10.1182/blood.2020009813 PMC8288657

[9] Victor Hoffbrand A , Hamblin TJ . Is “leukemia” an appropriate label for all patients who meet the diagnostic criteria of chronic lymphocytic leukemia? Leuk Res. 2007;31:273‐275. 16930694 10.1016/j.leukres.2006.07.006

[10] Montazeri A , Vahdaninia M , Harirchi I , Ebrahimi M , Khaleghi F , Jarvandi S . Quality of life in patients with breast cancer before and after diagnosis: an eighteen months follow‐up study. BMC Cancer. 2008;8:330. 19014435 10.1186/1471-2407-8-330 PMC2588619

[11] Reeve BB , Stover AM , Jensen RE , et al. Impact of diagnosis and treatment of clinically localized prostate cancer on health‐related quality of life for older Americans: a population‐based study. Cancer. 2012;118:5679‐5687. 22544633 10.1002/cncr.27578 PMC3410051

[12] Trevino KM , Martin P , Chen Z , Leonard JP . Worsening quality of life in indolent non‐Hodgkin lymphoma and chronic lymphocytic leukemia patients in active surveillance: a 12‐month longitudinal study. Clin Lymphoma Myeloma Leuk. 2022;22:82‐88. 34479847 10.1016/j.clml.2021.08.001 PMC8837721

[13] Shanafelt TD , Bowen D , Venkat C , et al. Quality of life in chronic lymphocytic leukemia: an international survey of 1482 patients. Br J Haematol. 2007;139:255‐264. 17897301 10.1111/j.1365-2141.2007.06791.x

[14] Dimopoulos MA , Voorhees PM , Schjesvold F , et al. Daratumumab or active monitoring for high‐risk smoldering multiple myeloma. N Engl J Med. 2025;392:1777‐1788. 39652675 10.1056/NEJMoa2409029

[15] Langerbeins P , Zhang C , Robrecht S , et al. The CLL12 trial: ibrutinib vs placebo in treatment‐naïve, early‐stage chronic lymphocytic leukemia. Blood. 2022;139:177‐187. 34758069 10.1182/blood.2021010845

[16] Hoechstetter MA , Wendtner CM . Clinical trials in early‐stage CLL: what has been learned and what's next? Leuk Lymphoma. 2025;66:378‐388. 39610305 10.1080/10428194.2024.2422839

[17] Oscier D , Dearden C , Eren E , et al. Guidelines on the diagnosis, investigation and management of chronic lymphocytic leukaemia. Br J Haematol. 2012;159:541‐564. 23057493 10.1111/bjh.12067

[18] Brieghel C , Galle V , Agius R , et al. Identifying patients with chronic lymphocytic leukemia without need of treatment: End of endless watch and wait? Eur J Haematol. 2022;108:369‐378. 35030282 10.1111/ejh.13743

[19] Shanafelt TD , Kay NE , Jenkins G , et al. B‐cell count and survival: differentiating chronic lymphocytic leukemia from monoclonal B‐cell lymphocytosis based on clinical outcome. Blood. 2009;113:4188‐4196. 19015397 10.1182/blood-2008-09-176149 PMC2676080

[20] Lea M , Hofmann BM . Dediagnosing—a novel framework for making people less ill. Eur J Intern Med. 2022;95:17‐23. 34417089 10.1016/j.ejim.2021.07.011

[21] Hibbs S , Van Blarikom E . Why do we diagnose monoclonal B‐cell lymphocytosis? Five questions. HemaSphere. 2023;7:e890. 37304932 10.1097/HS9.0000000000000890 PMC10256376

[22] Freeman T , Johnstone P , Hibbs SP , et al. Immunophenotyping for the assessment of asymptomatic lymphocytosis: a retrospective analysis and national survey. Eur J Haematol. 2025;114:303‐309. 39494776 10.1111/ejh.14336

